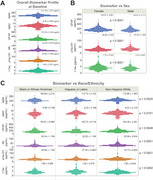# Baseline Biomarker Profiles in the Diverse Vascular Contributions to Cognitive Impairment and Dementia (DVCID) Study

**DOI:** 10.1002/alz70856_103380

**Published:** 2025-12-26

**Authors:** Sitong Zhou, Nopparat Suthprasertporn, Danielle J. Harvey, Charles Decarli, Myriam Fornage, Lee‐Way Jin

**Affiliations:** ^1^ University of California Davis Medical Center, Sacramento, CA, USA; ^2^ University of California, Davis, Sacramento, CA, USA; ^3^ University of California, Davis School of Medicine, Sacramento, CA, USA; ^4^ University of California, Davis, Davis, CA, USA; ^5^ The Brown Foundation Institute of Molecular Medicine, McGovern Medical School, The University of Texas Health Science Center at Houston, Houston, TX, USA

## Abstract

**Background:**

The DVCID project, funded by the National Institute of Neurological Disorders and the National Institute on Aging, aims to enroll 2250 diverse at‐risk Americans from diverse backgrounds with two follow‐up visits spaced 12 to 18 months apart. This comprehensive study integrates cognitive assessments, blood‐based analyses, and neuroimaging. Understanding baseline biomarker profiles is critical to the study's objectives, providing a foundation for future follow‐up measurements and enabling robust longitudinal analyses.

**Method:**

Blood samples from participants were collected, processed and aliquoted at baseline across all participating sites following standardized protocols. Plasma biomarker measurements were performed using the Quanterix Simoa HD‐X™ Analyzer by the Repository Core (RC) at University of California, Davis (UCD), quantifying amyloid beta 40 (Aβ40), Aβ42, glial fibrillary acidic protein (GFAP), neurofilament light chain (NfL), total Tau (t‐Tau), and phosphorylated tau181 (pTau181). Baseline data are presented; sex and racial/ethnic differences in biomarkers are also examined with t‐tests and ANOVA or non‐parametric versions, when appropriate.

**Result:**

Plasma biomarkers from the first 636 participants at baseline are quantified with the following concentrations (mean ± standard error): Aβ40 (113.18 ± 1.07 pg/mL), Aβ42 (6.30 ± 0.09 pg/mL), GFAP (157.82 ± 3.22 pg/mL), NfL (23.49 ± 0.62 pg/mL), pTau181 (30.88 ± 0.58 pg/mL) and t‐Tau (1.68 ± 0.03 pg/mL) as shown in Figure A. Significant sex differences are observed between 422 females and 214 males in GFAP, pTau181, and total Tau levels (*p* <0.0001) (Figure B). Plasma biomarker concentrations also vary across racial/ethnic groups (*p* <0.0001), with non‐Hispanic White participants (*N* = 295) presenting the highest levels of Aβ40, Aβ42, GFAP, NfL, and pTau181. Hispanic/Latino group (*N* = 181) exhibit the highest t‐Tau levels, while Black/African American group (*N* = 153) shows intermediate biomarker levels across all measured analytes (Figure C).

**Conclusion:**

The findings provide a strong foundation for future follow‐up studies. Observed sex and racial/ethnic differences in plasma biomarker levels emphasize the importance of considering demographic factors when interpreting biomarker data. Future investigation could focus on adjusting for age, BMI, HbA1C and white matter hyperintensity volume to explore the biological implications of these biomarkers in vascular dementia progression. This approach lays the groundwork for robust longitudinal analyses within the DVCID study.